# Factors Associated with Visual Fatigue from Curved Monitor Use: A Prospective Study of Healthy Subjects

**DOI:** 10.1371/journal.pone.0164022

**Published:** 2016-10-04

**Authors:** Haeng Jin Lee, Seong-Joon Kim

**Affiliations:** 1 Department of Ophthalmology, Seoul National University College of Medicine, Seoul, Republic of Korea; 2 Seoul Artificial Eye Center, Seoul National University Hospital Clinical Research Institute, Seoul, Republic of Korea; University of Illinois at Chicago, UNITED STATES

## Abstract

**Purpose:**

To investigate the factors associated with visual fatigue using monitors with various radii of curvature.

**Methods:**

Twenty normal healthy adults (8 men, 12 women; mean age, 26.2 ± 2.5 years) prospectively watched five types of monitors including flat, 4000R, 3000R, 2000R, and 1000R curved monitors for 30 min. An experienced examiner measured the ophthalmological factors including near point of accommodation (NPA), near point of convergence (NPC), refraction, parameters during pupil response at light and saccadic movement just before and after the visual tasks. The questionnaires about subjective ocular symptoms were also investigated just before and after the visual tasks.

**Results:**

The NPA increased after the visual tasks with a flat monitor compared with the curved monitors, with the 1000R curved monitor showing the smallest change (*p* = 0.020). The NPC increased for every monitor after the visual tasks; the largest increase occurred with the flat monitor (*p* = 0.001). There was no difference in refractive error, pupil response, or saccadic movement in the comparison of before and after the visual tasks. Among the nine factors in the questionnaire, the score of “eye pain” was significantly higher for the flat monitor versus the 1000R curved monitor after the visual tasks (*p* = 0.034).

**Conclusions:**

We identified NPA, NPC, and eye pain as factors associated with visual fatigue. Also, the curvature of the monitor was related to the visual fatigue.

## Introduction

Today, most people work at monitors for significant amounts of time, and many activities can be carried out without moving from monitors. There have been numerous health complaints associated with working at visual display terminals. Of these, eye problems are the single most common complaint.

Recently, studies have demonstrated a high prevalence of visual discomfort in video display terminal users [1–3]. Frequently reported complaints after using computers include eye strain, burning, tearing, irritation, redness, foreign body sensation, blurred vision, and double vision [4, 5]. Many of these symptoms have been proposed to be the result of the increased occurrence of dry eye syndrome [6–8]. Other studies also have reported reversible changes in visual function, such as reduced accommodation velocity, transient myopia, and reduced pupillary movement in the near reflex, suggesting possible effects on visual fatigue [9–11].

However, determining the prevalence of eye problems is difficult due to the vague nature of the complaints and the presence of numerous confounding factors. Also, asthenopia is a subjective visual symptom; thus, reliable objective parameters to quantify visual fatigue are still controversial. Furthermore, many new types of smart displays in addition to monitors are developed these days and there is a growing interest in visual fatigue. Therefore, clarification of the factors related to visual fatigue could be helpful to assess this growing problem.

Also, recently there were some asserts that the curved monitor would induce less visual fatigue than the flat monitor and actually curved monitors have been released and on sale. However, there has been no report on which device characteristics such as curvature or size affect the eyes.

In this study, we investigated the ophthalmological factors associated with visual fatigue and the effect of device characteristics on the visual fatigue using monitors with various radii of curvature.

## Materials and Methods

### Subjects

Study subjects included healthy volunteers recruited through an advertisement at Seoul National University Hospital, South Korea. The study protocol was approved by the Seoul National University Hospital Institutional Review Board and followed the tenets of the Declaration of Helsinki. All participants provided their written informed consent which IRB had approved.

The volunteers had no systemic or ocular disease and were aged between 20 and 30 years. They were tested for their distant and near visual acuity and underwent ophthalmological examinations, including intraocular pressure measurements, anterior segment examinations, and alternate prism cover tests. “Normal” eyes were defined as no past history of surgery for ocular disease, intraocular pressure less than 21 mmHg, spherical equivalent refraction less than ±3.0 diopters, astigmatism less than ±1.0 diopters, best-corrected visual acuity above 20/20, and with no abnormal findings in slit lamp biomicroscopy. Also, we requested the subjects to get enough sleep the day before the experiment and to visit at the same time on different days.

### Monitors and Visual Tasks

**Fig 1:**
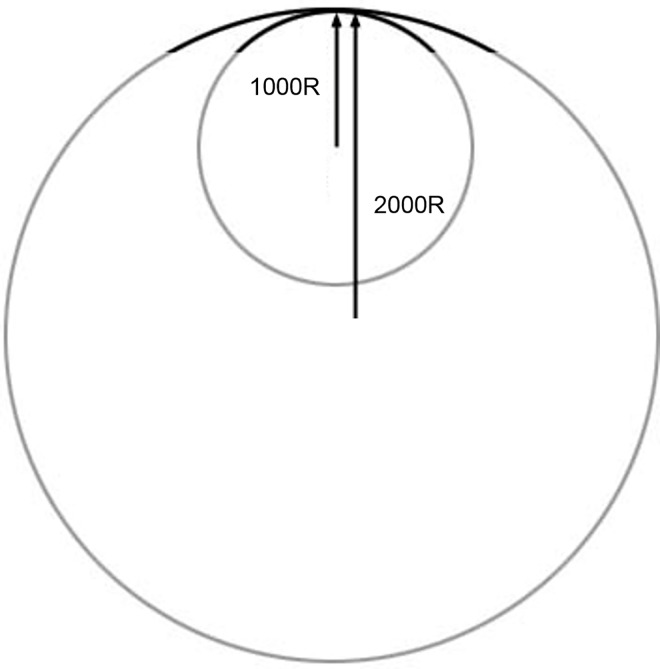
Example of the radius of curvature. A small (large) radius indicates a high (low) curvature. The 1000R curved monitor has higher curvature than the 2000R curved monitor.

There were five types of monitors with different curvature: flat, 4000, 3000, 2000, and 1000 mm radii of curvature (Fig 1). The size of the monitors was 32.3 inches in width and 14.3 inches in height with a resolution of 3440 × 1440 pixels (Samsung Electronics Co., Korea).

All subjects visited five times at the same time on different days to test all five types of monitors. One of the monitors was randomly assigned to the subjects. The random order of assigning monitors was based on William’s design [12]. The monitor was placed 70 cm from the eyes; subjects performed visual tasks for 30 min. The same examiner observed the subjects during the visual tasks to ensure a constant distance from the monitor. If subjects’ spherical equivalent refraction was over ±0.5 diopter, the optical correction was performed during the examination.

To induce visual fatigue, subjects performed intensive same visual tasks on all monitors. The visual tasks consisted of six tasks requiring subjects to concentrate on the monitors, including chasing fast or slow moving targets, finding big or small targets that appeared and disappeared suddenly, and reading long moving sentences to find an answer. All targets moved from the left side to the right side of the monitors or the opposite, which made subjects to watch not only the center but also the peripheral sides of monitors during the examination. After the visual tasks, we scored the examination. To make subjects concentrate fully on the visual tasks, we required them to score over 80%. If the score was under 80%, we planned to retest them another day.

### Ophthalmological Examinations

**Fig 2:**
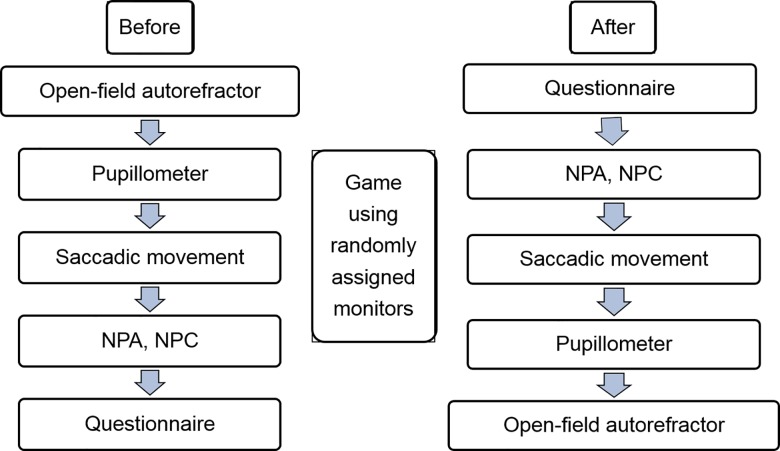
Experimental protocol. The objective and subjective factors were measured before and after watching monitors. (NPA: near point of accommodation; NPC: near point of convergence).

We examined only one eye of subjects by choosing the dominant eye using the hole-in-the-card test. The following procedures are noted in Fig 2. All examinations were performed by the same experienced examiner.

Refractive error was measured using an open-field autorefractor (N-vision-K5001, Shin-Nippon, Japan) at far, 50-, 40-, 33-, 25-, and 20-cm distances. A video-based infrared eye tracker device including a pupillometer (MonCv3, Metrovision, France) measured the pupil size under a mesopic environment; surrounding background room illumination was maintained as 4.0 lux and the lights in the monitor were changed as the 1cd/m^2^. The velocity, amplitude, and latency of pupil contraction and dilation during a light response were also measured. The saccadic velocity, amplitude, and latency during horizontal saccadic movement were also evaluated.

The near point of accommodation (NPA) was measured with the near-point ruler by push-up method. The test was performed on the dominant eye chosen by the hole-in-the-card test with the opposite eye occluded. Under room illumination, the subject first defined the smallest line of letters (printed on the square box) that could clearly be read at a distance of about 30 cm. Then, the target was moved towards the subject’s dominant eye, to the point at which the letters started to become ‘blurred.’ The distance from this point to the eye was then measured and recorded as centimeters.

In the near point of convergence (NPC) assessment, the subject was asked whether the identified target was seen as a single small round target when the examiner held it approximately 30 cm in front of the subject’s eyes. The examiner then moved the target slowly (~1–2 cm/s) towards the tip of the subject’s nose which is the center of two eyes. The endpoint was noted when the subject reported that the target appeared double or when the examiner saw one eye deviate from the target. The measurement was made after full correction of any refractive error with glasses if needed. To obtain reliable values, we fully explained these tests to the subjects and performed them several times before the experiment.

### Evaluation of Subjective Symptoms Using a Questionnaire

Subjects completed a questionnaire just before and after the visual tasks. We used a previously published questionnaire [13] containing nine identical analog scales on which the subject recorded the magnitude of each of the following nine symptoms: burning, eye pain, strain, irritation, tearing, blurred vision, double vision, dryness, and headache. Each analog scale was a 100-mm line with descriptors at both ends (0: none and 100: severe). The subject indicated magnitude with a vertical line along the scale, which was recorded as a value between 0 and 100.

### Statistical Analysis

Statistical analyses were performed using the SPSS software (ver. 16.0 for Windows; SPSS Inc., Chicago, IL, USA). For all tests, significance was set at a value of *p* < 0.05. Because all subjects performed the tasks five times at the same time on different days, the carryover and period effects could exist. The carryover effect is effect of previous examination on the next examination when repeated measurements were performed to the same subjects. The period effect is time interval for examination. Therefore, differences in factors were compared using a mixed regression model considering carryover and period effects. Also, we adjusted baseline value to compare all the ophthalmological factors between pre- and post-visual tasks.

## Results

### Subjects

In total, 20 volunteers were enrolled (8 men, 12 women). Their mean age was 26.2 ± 2.5 (range 21~30) years, and their mean base refractive error was -0.75 ± -1.25 (range -3.0 ~ +0.5) diopters. 11 subjects were assessed with right eye dominance and the others had left eye dominance. Also, the carryover and period effect were statistically insignificant in all measurements, which means that repeated measurements with time interval didn’t affect the results.

### Near Point of Accommodation

**Fig 3:**
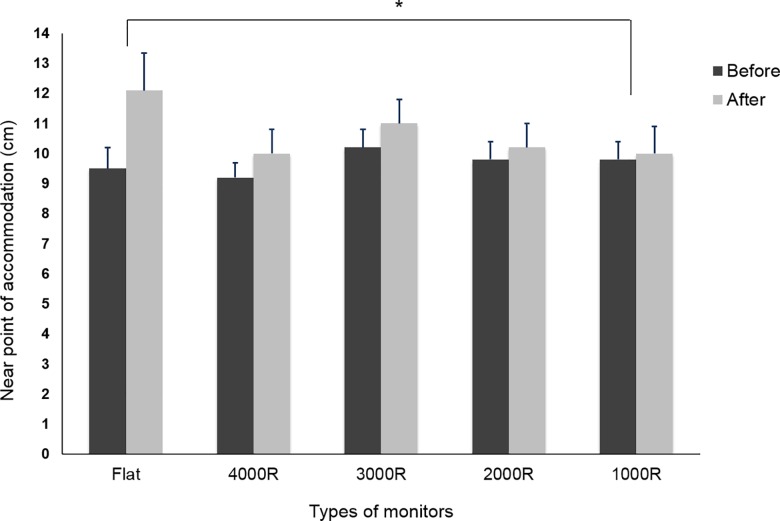
Changes in NPA between before and after the visual tasks. The NPA increased significantly after the visual tasks with the flat monitor. The 1000R curved monitor showed the smallest change (*p < 0.05).

The NPA was 9.5 ± 3.3 cm (mean 10.5 diopters) before the visual tasks and 12.1 ± 5.8 cm (mean 8.3 diopters) after the visual tasks with a flat monitor, which was a significant increase. However, there was no significant change with any of the curved monitors in a comparison of NPA values before and after the visual tasks. Especially, with the 1000R curved monitor, the NPA was 9.8 ± 2.6 cm (mean 10.2 diopters) before the visual tasks and 10.0 ± 4.2 cm (mean 10.0 diopters) after the visual tasks, showing the smallest change in NPA (*p* = 0.020) (Fig 3).

### Near Point of Convergence

**Fig 4:**
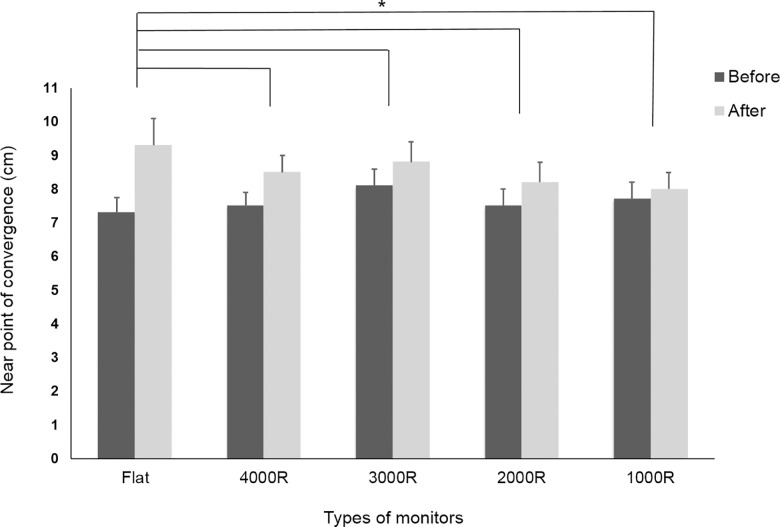
Changes in NPC before and after the visual tasks. The NPC increased significantly after the visual tasks with each monitor. The flat monitor showed the largest change in NPC (*p < 0.05).

The NPC before the visual tasks was 7.7 ± 2.2, 7.5 ± 2.3, 8.1 ± 2.6, 7.5 ± 2.3 and 7.3 ± 2.1 cm for the 1000R, 2000R, 3000R, 4000R, and flat monitors, respectively. The NPC was significantly higher for each monitor after the visual tasks: 8.0 ± 2.4, 8.2 ± 2.7, 8.8 ± 2.8, 8.5 ± 2.4, and 9.3 ± 3.7 cm for the 1000R, 2000R, 3000R, 4000R, and flat monitors, respectively. The changes in NPC after the visual tasks increased in the following order: 1000R, 2000R, 3000R, 4000R, and flat. The NPC increased most for the flat monitor as confirmed by the statistically significant difference between the flat and curved monitors (*p* = 0.001) (Fig 4).

### Refractive Error

The mean refractive errors before the visual tasks were +0.01, -1.06, -1.45, -1.95, -2.92, and -3.82 diopters for far, 50-, 40-, 33-, 25-, and 20-cm distances, respectively. After the visual tasks, the mean refractive errors were -0.10, -1.02, -1.52, -2.02, -2.86, and -3.87 diopters for far, 50-, 40-, 33-, 25-, and 20-cm distances, respectively. There was a mild myopic shift after the visual tasks at far, 40-cm, and 33-cm distances with all monitors. However, there was no significant difference among monitors (*p* = 0.334).

### Pupil Response

The mean pupil size before the visual tasks was 4.1 mm and 4.4 mm after the visual tasks. The amplitude of contraction was 1.8 mm both before and after the visual tasks. The velocity of contraction was 5.6 mm/s before the visual tasks, 5.7 mm/s after the visual tasks, and the velocity of dilation was 2.3 mm/s before the visual tasks, and 2.2 mm/s after the visual tasks. There was no significant change in all the pupil parameters during the light response in a comparison of values before and after the visual tasks with any types of monitors (*p* = 0.254) (Table 1).

**Table 1:** The pupil parameters during light response before and after the visual tasks.

	Pupil size (mm)		Contraction						Dilation			
			Amplitude (mm)		Latency (ms)		Velocity (mm/s)		Latency (ms)		Velocity (mm/s)	
	Before	After	Before	After	Before	After	Before	After	Before	After	Before	After
**Flat**	4.1	4.4	1.8	1.8	202.7	204.5	5.6	5.8	856.8	861.0	2.3	2.2
**4000R**	4.1	4.4	1.8	1.8	205.9	207.0	5.6	5.7	855.5	859.8	2.2	2.2
**3000R**	4.1	4.4	1.8	1.8	205.1	206.4	5.6	5.7	855.3	860.8	2.2	2.2
**2000R**	4.1	4.4	1.8	1.8	204.4	206.3	5.6	5.7	856.4	861.3	2.3	2.2
**1000R**	4.1	4.4	1.8	1.8	203.6	205.5	5.6	5.7	856.5	861.7	2.3	2.2

There was no significant change before and after the visual tasks in all types of monitors.

### Saccadic Movement

The mean velocity during the saccadic movement in all monitors was 19.7 ± 0.8 d/s before the visual tasks and 19.8 ± 0.4 d/s after the visual tasks. The mean latency during the saccadic movement in all monitors was 231.4 ± 4.0 ms before the visual tasks and 234.8 ± 5.3 md after the visual tasks. There was no significant change in saccadic mean velocity or latency in the comparison of before and after the visual tasks with any monitors (*p* = 0.721) (Table 2).

**Table 2:** The velocity during saccadic movements before and after the visual tasks.

		Center—Right		Right—Center		Center—Left		Left—Center	
		Before	After	Before	After	Before	After	Before	After
**Flat**	Velocity (d/s)	19.0	19.4	19.3	19.6	21.0	20.5	19.5	19.8
	Latency (ms)	229.2	240.7	233.1	231.4	238.7	242.2	230.8	235.2
**4000R**	Velocity (d/s)	18.8	19.3	19.3	19.5	20.9	20.4	19.4	19.7
	Latency (ms)	226.0	237.3	229.7	223.9	235.8	236.7	226.4	231.7
**3000R**	Velocity (d/s)	18.9	19.3	19.3	19.5	20.9	20.4	19.5	19.7
	Latency (ms)	226.9	238.6	230.4	225.0	237.0	237.1	228.2	232.5
**2000R**	Velocity (d/s)	18.9	19.4	19.3	19.6	20.9	20.5	19.5	19.8
	Latency (ms)	227.7	238.2	230.9	226.9	237.5	240.0	229.2	233.7
**1000R**	Velocity (d/s)	18.9	19.4	19.3	19.6	20.9	20.5	19.5	19.8
	Latency (ms)	229.1	239.3	232.0	229.1	238.7	241.4	230.0	234.6

There was no significant change in saccadic movements in all types of monitors.

### Questionnaire

The total subjective symptom score before the visual tasks was 47.3, 39.9, 40.9, 43.7, and 46.3 for the flat, 4000R, 3000R, 2000R, and 1000R monitors, respectively. After the visual tasks, total score was 89.9, 82.2, 83.5, 85.7, and 86.6 for the flat, 4000R, 3000R, 2000R, and 1000R monitors, respectively. It increased after the visual tasks with each monitor, and there was no difference according to the curvature of the monitors (*p* = 0.225). Among the nine factors in the questionnaire, the score of “eye pain” was significantly higher with the flat monitor versus the 1000R curved monitor; mean differences in eye pain (after value–before value) ± standard error of all 20 subjects were 10.4 ± 3.9, 10.3 ± 3.8, 10.4 ± 3.8, 10.3 ± 3.9, and 8.5 ± 2.0 for the flat, 4000R, 3000R, 2000R, and 1000R monitors, respectively. (*p* = 0.034) (Table 3).

**Table 3:** Changes in subjective symptom score before and after the visual tasks using questionnaire.

		Flat	4000R	3000R	2000R	1000R
**Eye strain**	Before	5.5	5.4	5.5	5.4	5.5
	After	12.5	12.5	12.7	12.5	12.6
**Eye pain**	Before	10.3	8.7	8.9	9.3	9.8
	After	20.7	19	19.3	19.6	18.3
**Headache**	Before	3.2	3.1	3.1	3.1	3.2
	After	7.7	7.8	7.8	7.7	7.7
**Double vision**	Before	2.9	2.2	2.4	2.8	2.9
	After	5.7	5	5.3	5.6	5.7
**Blurred vision**	Before	3.2	2.1	2.2	2.4	2.9
	After	5.2	4.1	4.1	4.3	5.0
**Irritation**	Before	5	4.1	4.2	4.8	5.0
	After	9	8.2	8.2	8.8	9.1
**Burning sensation**	Before	3.8	2.5	2.5	3.1	3.8
	After	6.1	4.4	4.5	5.0	5.7
**Dry eye**	Before	11.2	9.7	10	10.5	11.0
	After	19.6	17.9	18.2	18.8	19.3
**Tearing**	Before	2.2	2.2	2.2	2.2	2.2
	After	3.4	3.3	3.3	3.4	3.4
**Total score**	Before	47.3	39.9	40.9	43.7	46.3
	After	89.9	82.2	83.5	85.7	86.6

The total subjective symptom score was higher after the visual tasks for each monitor. The score for “eye pain” was significantly higher with the flat monitor versus that with the 1000R curved monitor.

## Discussion

Many people spend an increasing amount of time in front of computer screens; prolonged exposure can induce ocular discomfort, such as eye strain, burning sensation, and blurred vision [4, 5]. However, these eye problems are difficult to measure and clarify. We attempted to determine the ophthalmological factors related to visual fatigue.

Previous studies reported higher NPA after prolonged close work [14]. These changes could be induced by strong ciliary muscle contracture, or spasm of accommodation, referred to as ‘retarded relaxation,’ an inability to relax accommodation at a far distance in the normal rapid manner. These effects may be even greater if viewing durations are extended. In addition, these accommodative difficulties are considered to be among the main causes of asthenopia [14–16]. In this study, the NPA was higher after the visual tasks with the flat monitor; the 1000R curved monitor showed the smallest changes in NPA versus the flat monitor. The prolonged near work would affect the ability of accommodation like previous studies, and the monitor curvature could be the one of associated factors with visual fatigue.

NPC is known to be related to binocular function. Wee et al [17] reported that NPA and NPC were significantly altered after watching 3D displays versus baseline data and accommodation and binocular vergence are the predominant ophthalmological factors that may influence asthenopia [18]. In this study, similar to previous studies, prolonged near work induced visual fatigue; convergence decreased, thus NPC increased for each monitor after the visual tasks. The change in NPC after the visual tasks increased in the following order: 1000R, 2000R, 3000R, 4000R, and flat. The NPC showed the greatest increase with the flat monitor, and there was significant difference compared with all of the curved monitors which also shows the monitor curvature would be a possible factor related to the visual fatigue.

After a sustained period of near work, people have reported slightly blurred distance vision for several seconds or even minutes. The first investigation to assess near-induced transient myopia objectively was performed by Ehrlich [19]. After a continuous 2-h binocular near task at 20 cm, there were significant initial post-task myopic shifts (mean = 0.29 diopters); these myopic shifts did not decay to the pre-task baseline during the 1-h post-task distance vision assessment. Another study reported that this transient distance blur was related to increased transient myopia resulting from possible abnormalities related to the accommodative system [20]. In this study, there was a mild myopic shift after the visual tasks at far, 40-cm, and 33-cm distances with all monitors.

Regarding parameters related to the pupil response, Matsuda et al [9] reported that the pupillary near response was well preserved, even in elderly individuals, and the parameters of the pupillary response were good candidates for establishing common standards to evaluate the effects of 3D viewing. However, the relationships between visual fatigue and the parameters measured by the pupillometer, including the pupil size, velocity, amplitude, and latency of pupil contraction and dilation during a light response, have not yet been studied. In this study, we analyzed parameters related to the pupil response after viewing monitors; no significant change was observed in the pupil parameters assessed with any of the monitors. Even if the visual task was sufficient to make the subjects concentrate on the monitors and induce visual fatigue, the viewing time with the monitors could not be enough to make more definitive changes in these parameters.

Saccadic eye movements are characterized by rapid movements of the eyes. Mostly saccades are executed as reflex-like behavior. Detailed analysis of eye movements has become an important diagnostic tool in neurology for the assessment of brain function [21]. For example, changes in saccade characteristics due to brain lesions or dementia have been demonstrated, but there has been no report about the saccadic movement associated with near work or visual fatigue. In this study, we first analyzed the saccadic movements after watching various types of monitors; there was no significant change with any of the monitors under the conditions tested. We assumed that, like parameters related to the pupil response, the watch time with the monitors was not sufficient to make more definitive changes in saccadic eye movements.

Subjective factors are more difficult to measure or quantify than objective factors. To date, as subjective assessment methods, questionnaires have commonly been used to evaluate visual fatigue. To evaluate subjective visual symptoms, we used the questionnaire from a previously published report [13]. This questionnaire consists of 100-mm line analog scale with descriptors at both ends (0 = none and 100 = severe). The subject indicated magnitude with a vertical line along the scale, which was recorded as a continuous value between 0 and 100 by preventing selection bias caused by discrete value. They also divided the factors associated with asthenopia into external symptom factors and internal symptom factors. The external symptom factors consisted of burning, irritation, and dryness that seemed related to the ocular surface. The internal symptom factors included eye strain, ache, headache, double vision and blur, and they were higher than external symptom factors for accommodative stress, convergence, and mixed astigmatism conditions. In our study, total subjective symptom score was higher after the visual tasks for every monitor tested; there was no difference according to the curvature of the monitors. However, among nine factors in the questionnaire, the score for “eye pain,” one of internal symptom factors, increased significantly with the flat monitor versus the 1000R curved monitor; this indicated that the 1000R curved monitor induced less eye pain than the flat monitor. In a previous study, “eye strain” was also reported as a factor related to visual fatigue [13]. The meanings of “eye strain” and “eye pain” in English are clearly different. However, “eye strain” in Korean as used in this study has quite a more ambiguous nuance than “eye pain,” and this could make subjects choose more “eye pain” than “eye strain”.

Based on the results of objective and subjective parameters, curved monitors showed the least changes in NPA, NPC and the score for “eye pain” compared to flat monitor, and especially 1000R curved monitor showed smallest changes after the visual tasks. In curved monitor, both sides of monitor curve towards the subject, which makes the difference of distances between the subject and monitor smaller than flat monitor. This small difference would be the reason why the parameters related to the accommodation or convergence showed the smallest changes in 1000R curved monitor after the visual tasks. This is similar to the concept of horopter. Owing to the curvature of monitor, the retinal binocular disparity in the peripheral visual field is relatively decreased, which reduces blurring in the peripheral visual field. There may be unknown brain signals leading to the subjects feeling more comfortable when watching a curved monitor. However, there have been no previous studies on the effects of monitor curvature. The reasons for the significant difference should be investigated further.

This study has several limitations. First, the viewing time with the monitors was about 30 min. A longer watch time may have caused more definitive changes, facilitating comparison of ocular factors. However, it is difficult for the same subject to focus on the monitors for more than 30 min at least five times. Furthermore, the task was enough to make the subjects concentrate on the monitors and to induce visual fatigue. Second, a double-blind examination was impossible, because both the examiner and subjects could be aware of which display they watched. Even if we randomly assigned the monitors to minimize this bias, there could be a minimal effect. Third, we conducted this study on healthy subjects and used only one brand of monitor that is 32 inches wide. In addition, some healthy subjects performed the tasks with optical correction. There could be minification effect of eyeglasses that affects contrast sensitivity. Depending on the brand and width of the monitor and on subjects having some forms of ocular disorders, the results could vary. Therefore, there are needs for the further investigation considering these factors.

Even though we could not find a new parameter to measure the visual fatigue, we found some objective factors related to visual fatigue. Especially, this study was performed under well-controlled settings, including a largely homogenous normal group of young subjects (mean age, 26.2 ± 2.5 years). In addition, we identified monitor curvature as a possible associated factor with visual fatigue.

In conclusion, we found that NPA, NPC, eye pain, and the curvature of the monitor were related to the visual fatigue. As the prevalence of asthenopia is expected to rise with increasing monitor use, more population-based studies with larger numbers of subjects are needed to confirm these results.
